# SPI-1 is a missing host-range factor required for replication of the attenuated modified vaccinia Ankara (MVA) vaccine vector in human cells

**DOI:** 10.1371/journal.ppat.1007710

**Published:** 2019-05-30

**Authors:** Ruikang Liu, Jorge D. Mendez-Rios, Chen Peng, Wei Xiao, Andrea S. Weisberg, Linda S. Wyatt, Bernard Moss

**Affiliations:** Laboratory of Viral Diseases, National Institute of Allergy and Infectious Diseases, National Institutes of Health, Bethesda, Maryland, United States of America; Ludwig-Maximilians-Universität München, GERMANY

## Abstract

Modified vaccinia virus Ankara (MVA) is the leading poxvirus vector for development of vaccines against diverse infectious diseases. This distinction is based on high expression of proteins and good immunogenicity despite an inability to assemble infectious progeny in human cells, which together promote efficacy and safety. Nevertheless, the basis for the host-range restriction is unknown despite past systematic attempts to identify the relevant missing viral gene(s). The search for host-range factors is exacerbated by the large number of deletions, truncations and mutations that occurred during the long passage history of MVA in chicken embryo fibroblasts. By whole genome sequencing of a panel of recombinant host-range extended (HRE) MVAs generated by marker rescue with 40 kbp segments of vaccinia virus DNA, we identified serine protease inhibitor 1 (SPI-1) as one of several candidate host-range factors present in those viruses that gained the ability to replicate in human cells. Electron microscopy revealed that the interruption of morphogenesis in human cells infected with MVA occurred at a similar stage as that of a vaccinia virus strain WR SPI-1 deletion mutant. Moreover, the introduction of the SPI-1 gene into the MVA genome led to more than a 2-log enhancement of virus spread in human diploid MRC-5 cells, whereas deletion of the gene diminished the spread of HRE viruses by similar extents. Furthermore, MRC-5 cells stably expressing SPI-1 also enhanced replication of MVA. A role for additional host range genes was suggested by the restoration of MVA replication to a lower level relative to HRE viruses, particularly in other human cell lines. Although multiple sequence alignments revealed genetic changes in addition to SPI-1 common to the HRE MVAs, no evidence for their host-range function was found by analysis thus far. Our finding that SPI-1 is host range factor for MVA should simplify use of high throughput RNAi or CRISPR/Cas single gene methods to identify additional viral and human restriction elements.

## Author summary

Poxvirus vectors have outstanding properties for development of vaccines against a myriad of infectious agents due to their ability to retain long segments of foreign DNA and high-level gene expression. Safety concerns led to a preference for attenuated poxviruses that lost the ability to produce infectious progeny in human cells. The most widely used poxvirus vector is modified vaccinia virus Ankara (MVA), which exhibits an extreme host-range restriction in most mammalian cells. MVA was attenuated by passaging more than 500 times in chicken embryo fibroblasts during which large deletions and numerous additional genetic changes occurred. Despite ongoing clinical testing of MVA-vectored vaccines, the basis for its host-range restriction remained unknown. Here we show that re-introduction of the SPI-1 gene into MVA or host cells increased virus spread by more than 100-fold in a human diploid cell line, providing an important insight into the mechanism responsible for the host-range restriction. This information could help design improved vectors and develop non-avian cell lines for propagation of candidate MVA vaccines.

## Introduction

Vaccinia virus (VACV) has been developed as a live recombinant expression vector that is widely used for making candidate vaccines against unrelated pathogens [1–5]. Although VACV was successfully used as a smallpox vaccine, concerns regarding safety with regard to the creation of new vaccines led to interest in more attenuated poxvirus vectors including fowlpox virus [6], canarypox virus [7, 8], and recombinant VACV strains in which one or multiple genes were deleted selectively [9, 10] or by blind passaging [11, 12]. One such attenuated strain, modified vaccinia virus Ankara (MVA), was produced by passaging the parental chorioallantois vaccinia virus (CVA) strain more than 500 times in chicken embryo fibroblasts (CEF) for the purpose of producing a safe smallpox vaccine [11]. Initial analysis of the MVA genome revealed six major deletions compared to the parent virus [13]. These large deletions as well as numerous additional genetic changes were confirmed by genome sequencing [14]. Notwithstanding the loss of considerable genetic material and the consequent inability to efficiently produce infectious virus in most mammalian cells [13, 15–17], MVA retains the ability to express viral as well as recombinant proteins regulated by VACV promoters in non-permissive cells at levels comparable to replicating VACV and to induce both humoral and cellular immune responses [18, 19]. These beneficial features propelled the use of MVA for development of numerous candidate vaccines, some of which are in clinical trials [20].

Despite extensive testing of candidate MVA vaccines in humans, the basis for the host-restriction of MVA, which is important to fully understand its attenuation, remains unknown. The large number of deletions, truncations and mutations that occurred during the long passage history of MVA in CEF severely complicates efforts to determine those changes important for its host-range defect. Indeed, a comparison of MVA with its parent CVA revealed 71 orthologous ORFs predicted to encode identical gene products, whereas the remaining 124 ORFs encode gene products with amino acid changes, insertions or deletions [21]. One attempt to investigate the genetic changes responsible for the replication defect consisted of deleting DNA sequences corresponding to the six major deletions of MVA from the genome of the parental CVA [22]. Remarkably, the loss or truncation of 31 open reading frames (ORFS) totaling ~ 25 kbp of DNA from the parental virus was insufficient to produce the host-range phenotype of MVA, leading to the conclusion that the major determinants lie outside of these deletions. In a related approach, the large deletions of MVA were introduced into the Lister strain of VACV [23]. Loss of the genes corresponding to those missing from deletion I of MVA, located near the left end of the genome, reduced replication in HeLa cells by 4- to 5-fold. No additional effect was observed upon introducing the additional deletions of MVA into the Lister strain. A totally different approach entailed a marker rescue scheme in which recombinant viruses were produced by transfecting MVA-infected cells with cosmids containing DNA segments of ~40 kbp spanning the genome of a replicating strain of VACV [24]. When the infected cell lysates were plated on BS-C-1 cells, large plaques were observed in samples that had been transfected with DNA derived from the left end of the genome. Following clonal isolation, six of eight independently isolated recombinant viruses (v44.1, v44.2, v44/47.1, v44/47.2, v51.1, v51.2), named after the cosmids used for their rescue, were found to also replicate to high titers in human MRC-5, HeLa and A549 cells. We refer to these recombinant viruses as host-range extended (HRE) MVAs. A subsequent study demonstrated that v44/47.1 and v51.1 replicate well in monkey Vero cells, which are frequently used for vaccine manufacturing, while still exhibiting severe attenuation in immunocompetent and immunodeficient mice [25]. Thus far, there has been only a limited investigation of how the HRE MVAs overcome the host range restriction. Dobson and Tscharke [26] found that the F5L gene, which was restored in v44.1, was important for plaque morphology but did not enhance replication of MVA.

A comparison of the whole genome sequences of MVA and its parent CVA revealed that only two known host range genes, C12L and K1L, located near the left end of the genome, are specifically missing or truncated in MVA [21]. However, introduction of the K1L gene into MVA did not reverse the human host-range defect [27]. A corresponding study of the effects of insertion of the C12L gene had not been reported, even though polymerase chain reactions (PCR) of the HRE MVA genomes revealed a correlation of the acquisition of C12L DNA with replication in human cells [24]. The protein encoded by C12L belongs to the serine protease inhibitor superfamily known as serpins and is called serine protease inhibitor-1 (SPI-1) [28]. SPI-1 is conserved in orthopoxviruses and expressed as an intracellular non-glycosylated 40-kDa species [29]. Deletion of the SPI-1 ORF from rabbitpox virus (RPXV) or VACV strain WR causes diminished replication in human A549 and pig kidney 15 cells but not in several avian and monkey cell lines [29, 30]. A recent human genome-wide RNAi screen implicated three genes (IRF2, FAM111A and RFC3) in the restriction of SPI-1 deletion mutants in human A549 cells, although the mode of their action remains to be determined [31].

The primary intent of the present study was to identify specific genes lost during the passage history of MVA that contribute to its host-range defect. The whole genome sequences of five independently isolated HRE MVAs revealed the presence of an intact C12L open reading frame (ORF) in those viruses that gained the ability to replicate in human cells. Additionally, we observed a similar assembly block in MVA and a SPI-1 mutant derived from VACV WR. Most importantly, we demonstrated that insertion of the C12L ORF into MVA enhanced replication by more than 2 logs in human MRC-5 cells whereas deletion of the C12L ORF from the HRE MVAs diminished replication by similar amounts. Although multiple sequence alignments revealed additional genetic changes common to the HRE MVAs, no evidence for their host-range function has been found by mutational analysis thus far.

## Results

### Comparison of the morphogenesis block of MVA and a VACV WR SPI-1 deletion mutant

**Fig 1:**
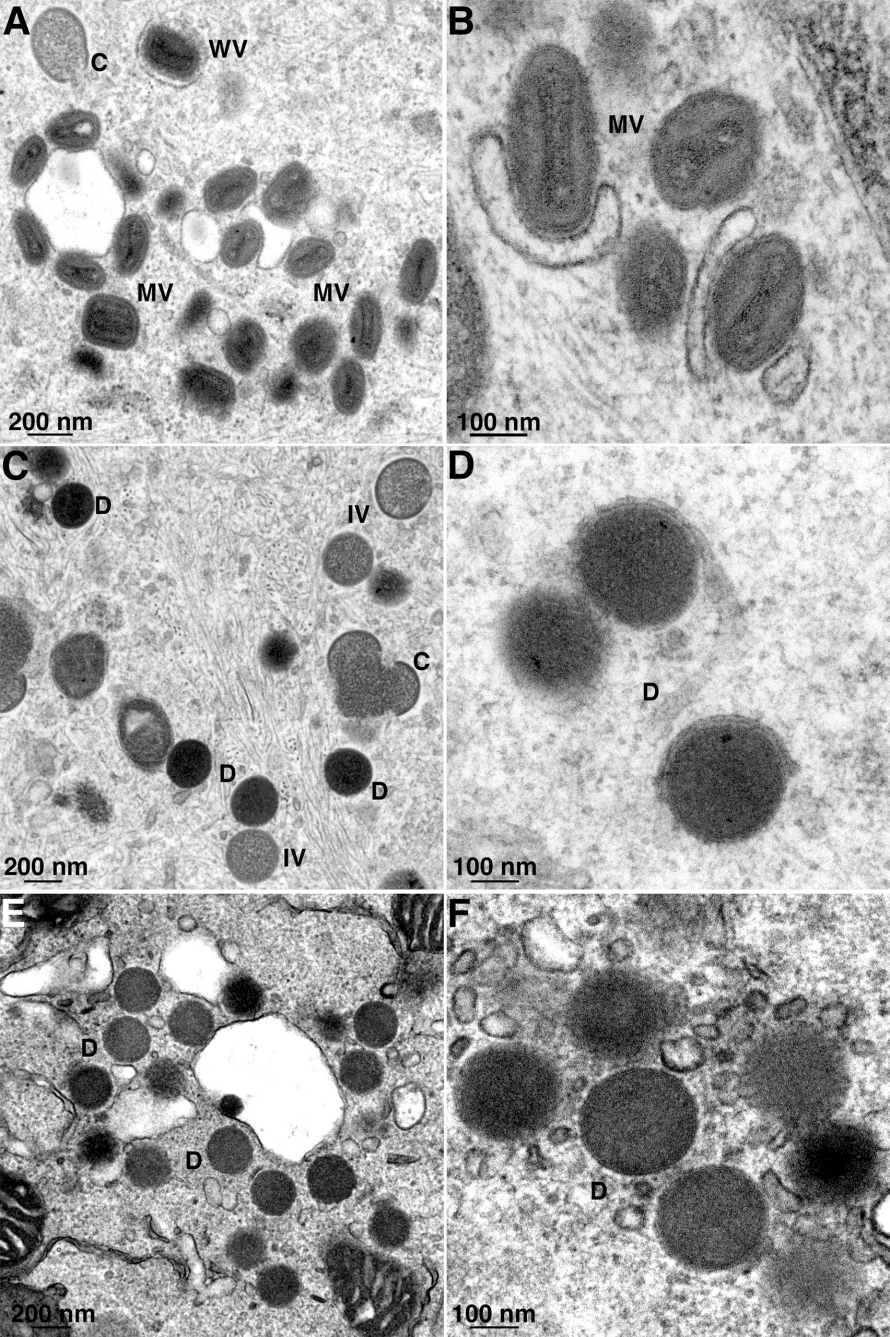
Morphogenesis defects of WRΔSPI-1 and MVA. A549 cells infected with VACV WR (**A, B**), WRΔSPI-1 (**C, D**) or MVA (**E, F**) were analyzed by transmission electron microscopy. Abbreviations: MV, mature virion; WV, wrapped virion; C, crescent; IV, immature virion; D, dense virion. Magnification shown below each panel.

Although numerous genes were deleted or truncated during the long passage history of MVA, the only ones with known human host-range function are C12L encoding SPI-1 and K1L. Table 1 summarizes previous PCR data [24] confirming the absence of C12L DNA and truncation of K1L in MVA. Strikingly, C12L was detected by PCR in all HRE MVAs that were able to replicate in human cells, whereas the presence of full-length K1L correlated with replication only in rabbit kidney cells (Table 1). The correlation of C12L and replication in human cells focused our attention on SPI-1 as a missing host-range factor for MVA. Unlike most orthopoxvirus host-range mutants, which exhibit blocks in gene expression, the earliest recognized defect in MVA replication occurs during morphogenesis [15, 18, 32]. Interestingly, the second exception to the general rule for the predominance of impaired gene expression is the morphogenesis defect of SPI-1 deletion mutants of rabbitpox virus and the WR strain of VACV in non-permissive cells [30, 33]. The possibility that the absence of the SPI-1 gene contributes to the host-range defect of MVA persuaded us to compare their morphogenesis blocks. Human A549 cells that had been infected for 24 h with VACV WR, a WR SPI-1 deletion mutant (WRΔSPI-1) or MVA were prepared for transmission electron microscopy. In the cells infected with WR (Fig 1A), there was a predominance of brick-shaped mature virions (MVs) and some wrapped or partially wrapped virions (WVs) as well as crescents (C) and immature virions (IVs). The cells infected with WRΔSPI-1 (Fig 1C) had many aberrant particles with the spherical shape and dimensions of IVs but with dense unstructured interiors, which are referred to as dense virions (DVs). Many spherical DVs were also present in the cells infected with MVA (Fig 1E). Higher magnification confirmed the similar appearances of the DVs in the cells infected with MVA and the WRΔSPI-1 (Fig 1D and 1F) and the more mature morphology of MVs in the cells infected with WR (Fig 1B). Thus, the impairment in morphogenesis occurred at a similar stage in non-permissive cells infected with MVA and WRΔSPI-1. Nevertheless, this similarity only provided suggestive evidence of related defects.

**Table 1:** Correlation of C12L and K1L with host range in human and rabbit cells^a^.

Virus^b^	C12L	K1L	Monkey cells	Human cells	Rabbit kidney cells
MVA	-	truncated	-	-	-
v51.1	+	+	+	+	+
v51.2	+	truncated	+	+	-
v44.1	+	truncated	+	+	-
v44.2	+	+	+	+	+
v47.1	-	truncated	+	-	-
v47.2	-	truncated	+	-	-
v44/47.1	+	+	+	+	+
v44/47.2	+	+	+	+	+

^a^Ability to replicate better than MVA (+) or equivalent to MVA (-).

^b^Viruses are named according to the cosmid or cosmids used for marker rescue and the

decimals indicate separate clones. Data are summarized from Wyatt et al. [24].

### Restoration of the SPI-1 gene increases MVA replication in human cells

The presence of the C12L ORF in HRE MVAs that replicate in human cells and the similarity in the morphogenesis block of MVA and other SPI-1 deletion mutants led us to investigate whether the introduction of the SPI-1 ORF into the MVA genome would have a discernible effect on replication in human cells. To facilitate the construction of the recombinant MVA, we used a transfer plasmid vector in which the C12L ORF was regulated by the well characterized modified mH5 promoter that has strong early and moderate late activities [34, 35]. Recombination occurred into the site of deletion III located near the right end of MVA so as not to interrupt or alter additional genes. Permissive CEF were used for infection and transfection and the recombinant virus, named MVA-SPI-1, was clonally isolated by several rounds of picking foci that fluoresce due to co-expression of the green fluorescent protein (GFP). PCR and Sanger sequencing were performed to confirm insertion of the complete C12L ORF.

**Fig 2:**
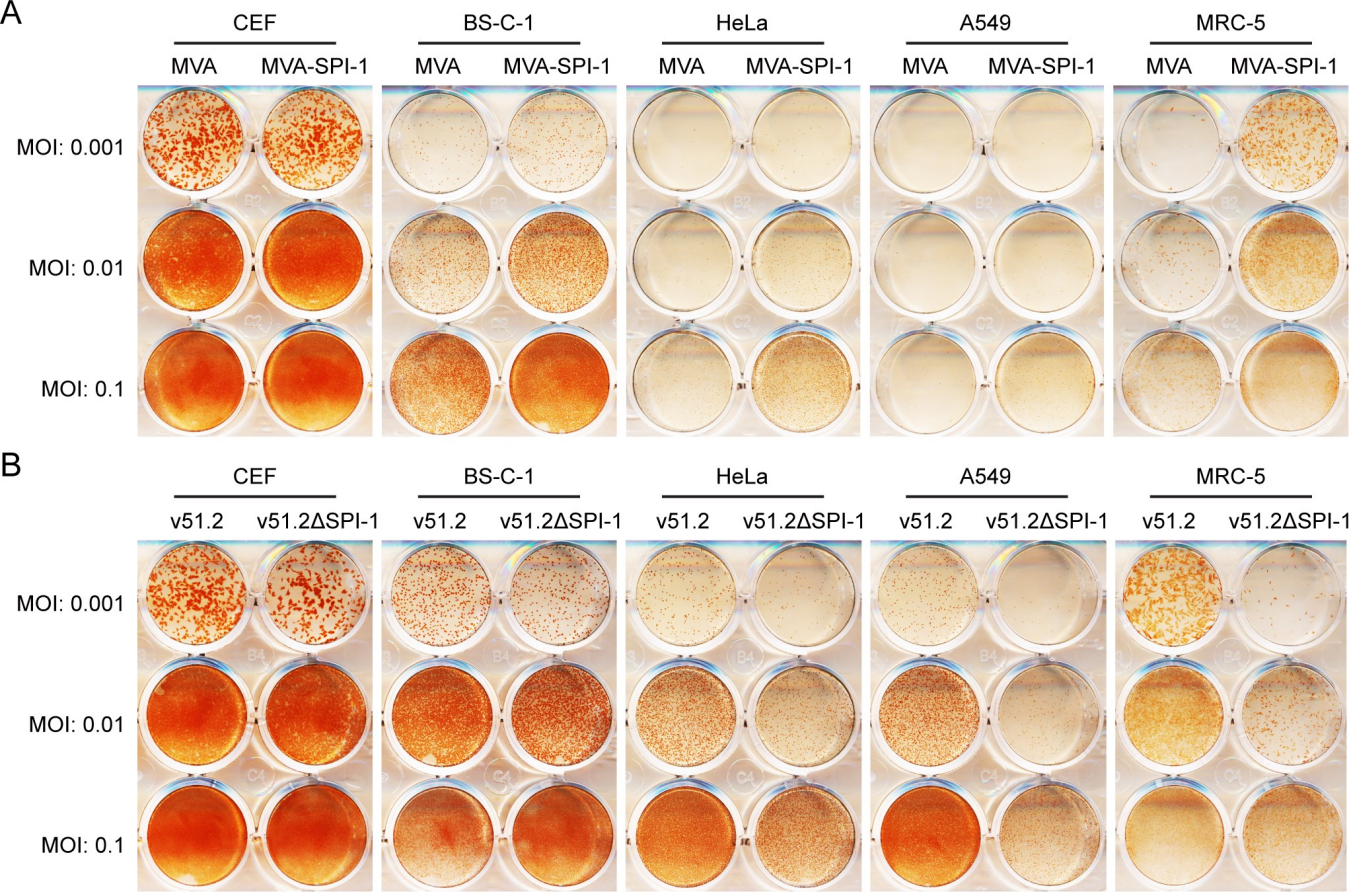
Requirement of SPI-1 for spread of MVA in human cells. **(A)** Effect of addition of the SPI-1gene to MVA. CEF, BS-C-1, HeLa, A549 and MRC-5 cells in 12-well plates were infected with MVA or MVA-SPI-1 at MOI of 0.001, 0.01 and 0.1 and overlaid with methylcellulose. After 48 h, the overlay was removed and cells were stained with antibody to VACV. **(B)** Effect of SPI-1 gene deletion on v51.2 spread. CEF, BS-C-1, HeLa, A549 and MRC-5 cells in 12-well plates were infected with v51.2 or v51.2ΔSPI at MOI of 0.001, 0.01 and 0.1 and overlaid with methylcellulose. After 48 h, the overlay was removed and cells were stained with antibody to VACV.

The effect of the C12L ORF addition was assessed by virus spread in several cell lines with a range of virus multiplicities of MVA or MVA-SPI-1. MVA is less cytopathic than other strains of VACV and does not form regular shaped plaques under semisolid medium that can be easily discerned by staining with crystal violet or neutral red. Consequently, the irregular foci formed were visualized by immunostaining with antibody to VACV. We used a range of virus multiplicities because of cytopathic effects that occur during the 48 h incubations. In permissive CEF, the two viruses formed foci of similar number, size and staining intensity best seen at a MOI of 0.001 before confluence occurred (Fig 2A). In monkey BS-C-1 and human MRC-5 cells, MVA-SPI-1 foci that were larger and exhibited more intense staining relative to MVA were best seen at MOI of 0.001 and 0.01 (Fig 2A). In HeLa and A549 cells, MVA-SPI-1 also exhibited an increase in staining relative to MVA, but the effect was less than in BS-C-1 and MRC-5 cells and was best discerned at a MOI of 0.1 (Fig 2A).

### Deletion of the SPI-1 gene from HRE MVAs reduces replication in human cells

To complement the results of addition of the SPI-1 gene to MVA, we deleted the gene from the HRE MVAs. This was accomplished by homologous recombination with DNA containing the GFP ORF regulated by the p11 late promoter within C12 flanking sequences. Recombination was carried out in CEF and the virus in fluorescent foci were clonally purified by repeated isolations. The loss of the C12L gene was confirmed by PCR and Sanger sequencing. The effect of the gene deletion from v51.2 (v51.2ΔSPI-1) was determined by infecting cells with 0.001 to 0.1 PFU per cell. There was no discernible effect of the gene deletion in CEF and only a slight effect in BS-C-1 cells, whereas in HeLa, A549 and MRC-5 cells the foci formed by v51.2ΔSPI-1 stained less intensely than those formed by the parent virus v51.2 (Fig 2B). A comparison of Fig 2A and 2B suggested that loss of SPI-1 by v51.2 had a greater impact than gain of SPI-1 by MVA particularly in A549 cells.

**Fig 3:**
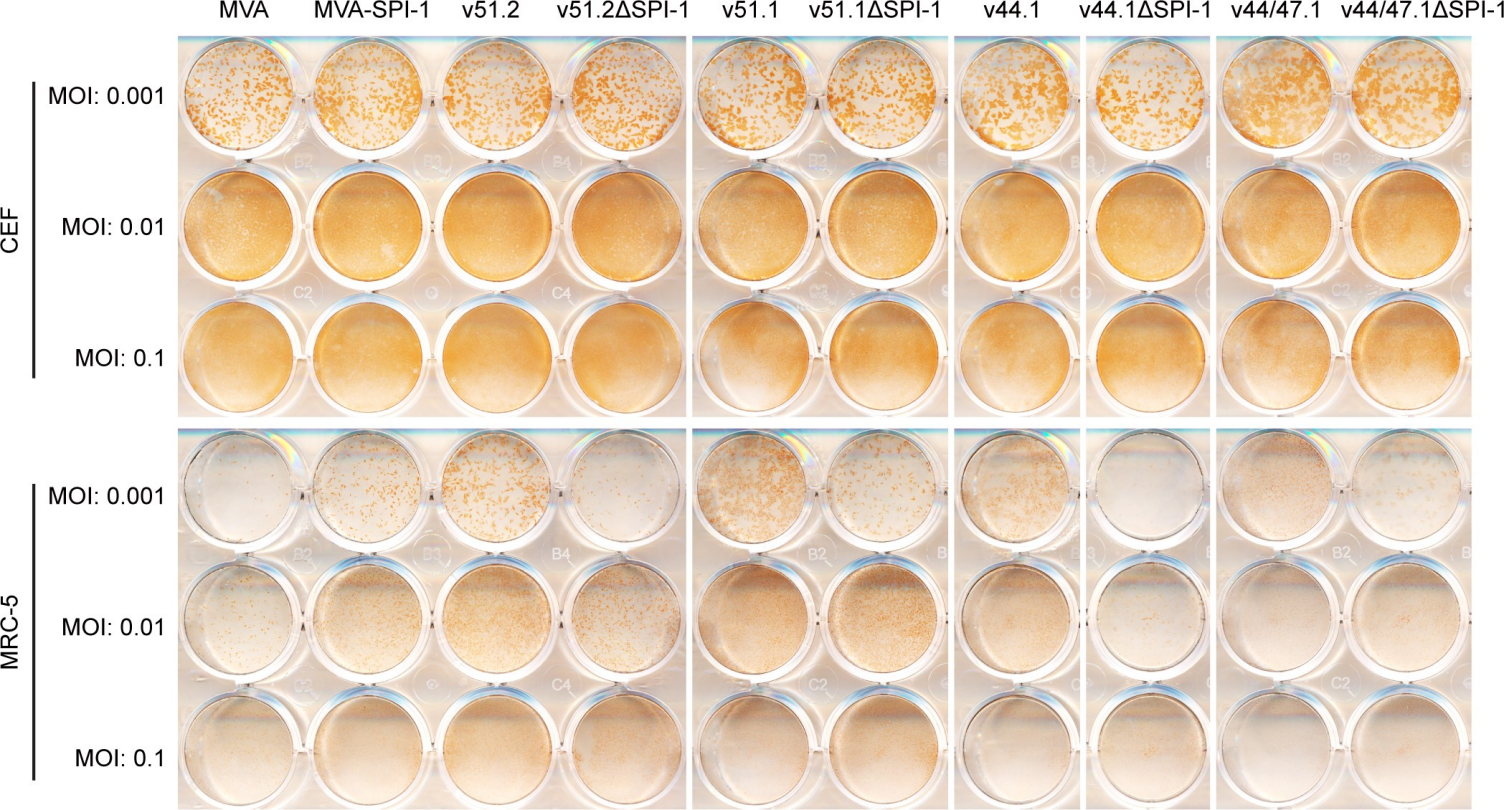
Effect of SPI-1 deletions on spread of additional HRE MVAs. CEF and MRC-5 cells in 12-well plates were infected with the indicated viruses at MOI of 0.001, 0.01, 0.1 and overlaid with methylcellulose. After 48 h, the overlay was removed and cells were stained with antibody to VACV.

We also compared the effects of SPI-1 deletions on the other independently isolated HRE MVAs (v51.1, v44.1 and v44/47.1). Although deletion of the C12L ORF had no discernible effect in CEF, in each case the staining intensity of foci was reduced in MRC-5 cells, which was most clearly seen at the lowest MOI (Fig 3).

### Effects of addition and deletion of SPI-1 on virus yields

**Fig 4:**
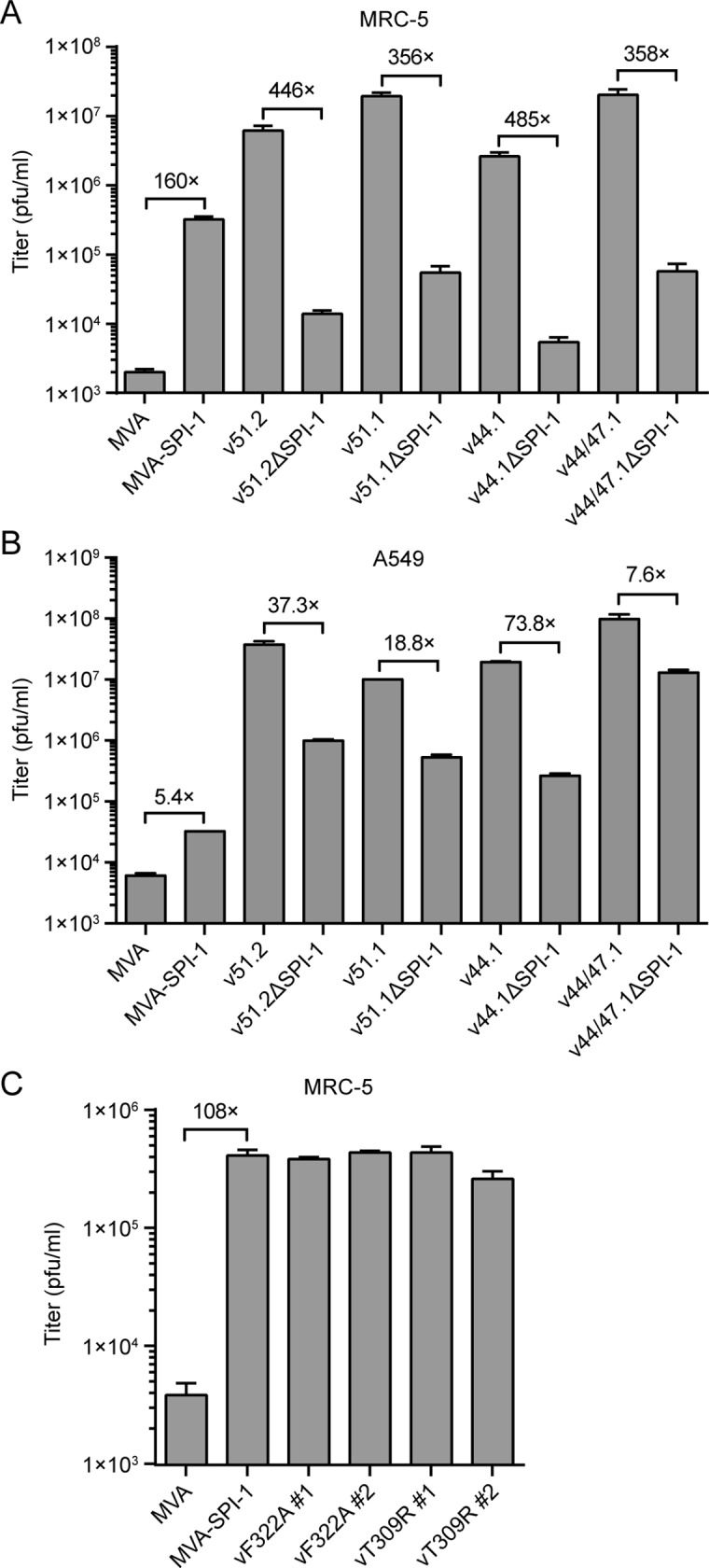
Effect of SPI-1 insertions and deletions on yields of recombinant MVAs. MRC-5 **(A)** or A549 (**B)** cells were infected in triplicate with indicated viruses at a MOI of 0.001 (MRC-5) or 0.01 (A549) cells for 48 h. Virus titers were determined in duplicate on CEF. **(C)** MRC-5 cells were infected as in panel A with MVA-SPI-1 or MVA recombinants containing F322A or T309R mutations in the SPI-1 ORF. Error bars indicate SEM and fold differences in titers are indicated.

Virus yields were determined at 48 h after inoculating MRC-5 and A549 cells with viruses at a multiplicity of infection (MOI) of 0.001 or 0.01 in order to quantify the effects of SPI-1 on replication and spread. In MRC-5 cells, expression of SPI-1 increased the virus yield relative to MVA by 160-fold (p = 0.0006) and deletion of SPI-1 from v51.2, v51.1, v44.1 and v44/47.1 reduced the yields by approximately 400-fold in each case (p<0.01) (Fig 4A). In A549 cells, there was also an increase in yield produced by expression of SPI-1 relative to MVA and a decrease in yield of HRE viruses due to deletion of SPI-1 (Fig 4B). However, both effects were much smaller than in MRC-5 cells. However, even in MRC-5 cells, addition of SPI-1 to MVA did not increase the yield to the levels of the HRE viruses, which all have C12L, nor did deletion of C12L from the latter viruses reduce the yield to the level of MVA. Therefore, we concluded that absence of SPI-1 strongly contributes to the host-range defect of MVA but is not the sole factor responsible.

Serine protease inhibitor activity of RPXV SPI-1 was suggested by the formation of a stable complex with cathepsin G *in vitro*, which was prevented by mutation of the phenylalanine to alanine in the putative reactive loop [36]. Furthermore, when the phenylalanine to alanine mutation was introduced into the RPXV genome, the host range was similar to that of an SPI-1 deletion mutant in A549 cells. However, we found that, recombinant MVAs containing SPI-1 with or without the reactive loop mutation (F322A) or a control mutation outside of the loop (T309R) enhanced MVA spread similarly in MRC-5 cells (Fig 4C) suggesting that SPI-1 may have more than one mode of function.

### Effects of ectopic expression of SPI-1 on host range

**Fig 5:**
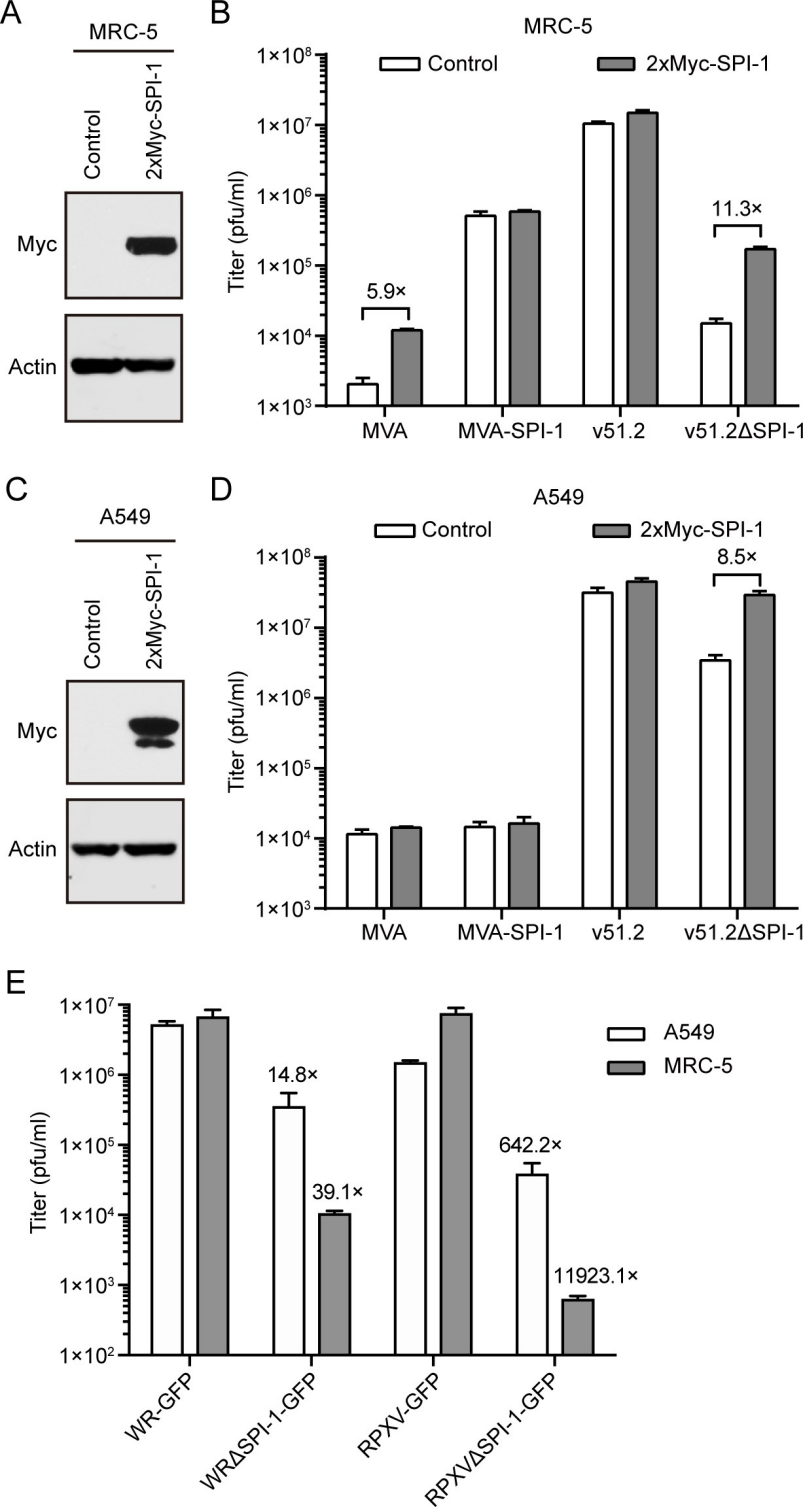
Replication of recombinant MVA, VACV WR, and RPXV in parental or SPI-1-expressing MRC-5 and A549 cells. (**A, C**) Expression of SPI-1. MRC-5 (A) and A549 (C) cells were infected with retroviruses that express 2xMyc-SPI-1 or control retroviruses and selected by antibiotic resistance. Western blots with antibody to the Myc tag are shown. The minor Myc band in panel C is assumed to be due to degradation. (**B, D**) Replication of MVA, MVA-SPI-1, v51.2, v51.2ΔSPI-1 at MOI of 0.01 in control and SPI-1 expressing MRC-5 (B) and A549 (D) cells. (**E**) A549 and MRC-5 cells were infected at a MOI of 0.01 in triplicate with VACV WR expressing GFP without (WR-GFP) or with a deletion of SPI-1 (WRΔSPI-1-GFP) and RPXV expressing GFP without (RPXV-GFP) or with a deletion of SPI-1 (RPXVΔSPI-1-GFP). In panels B, D and E the cells were infected in triplicate for 48 h and titers were determined in duplicate on CEF. Error bars represent SEM and fold difference in titers are indicated.

Thus far, we have shown that expression of SPI-1 by MVA and HRE MVAs enhanced replication in human cells. However, viral genome alterations could have unanticipated effects. To circumvent this potential problem, we determined the effect of trans-expression of SPI-1 on MVA replication. The C12L ORF with a 2xMyc tag regulated by the CMV promoter was introduced into A549 and MRC-5 cells by transduction with a retroviral vector. Expression of SPI-1 was demonstrated by Western blotting (Fig 5A and 5C). We would expect that ectopic expression of SPI-1 would have little or no effect on the replication of MVA-SPI-1 and v51.2 as they already express SPI-1, whereas there would be enhancement of MVA and v51.2ΔSPI-1. This is precisely what occurred in MRC-5 cells, with increases of approximately 6- and 11-fold for MVA and v51.2ΔSPI-1, respectively (Fig 5B). Ectopic expression of SPI-1 in A549 cells also increased replication of v51.2ΔSPI-1 (Fig 5D) but had little or no effect on replication of MVA. The latter result was consistent with the small enhancement of MVA-SPI-1 replication compared to MVA in A549 cells (Fig 4B). Thus, both trans- and cis-expression of SPI-1 have similar effects on host range.

### VACV WR and RPXV exhibit a requirement for SPI-1 in MRC-5 cells similar to MVA

We were curious whether the greater effects of addition and deletion of SPI-1 in MRC-5 cells compared to A549 cells was specific for MVA. To our knowledge, A549 is the only human cell line in which the effect of SPI-1 deletion had been tested for either RPXV or VACV WR [29–31]. For comparison, A549 and MRC-5 cells were infected with VACV WR and RPXV SPI-1 deletion mutants and the parental viruses. In A549 cells, deletion of SPI-1 reduced the spread of VACV WR and RPXV by 15-and 640-fold respectively, whereas in MRC-5 cells the reductions were 39- and 12,000 respectively (Fig 5E). Thus, not only was RPXV more dependent than VACV WR on SPI-1, but the requirement was greatly increased in MRC-5 cells compared to A549 cells for both viruses. We conclude that viral as well as cellular genetic backgrounds determine the degree of dependency on SPI-1 for replication.

### Whole genome sequences of HRE MVAs and effects of deletion of additional genes

**Fig 6:**
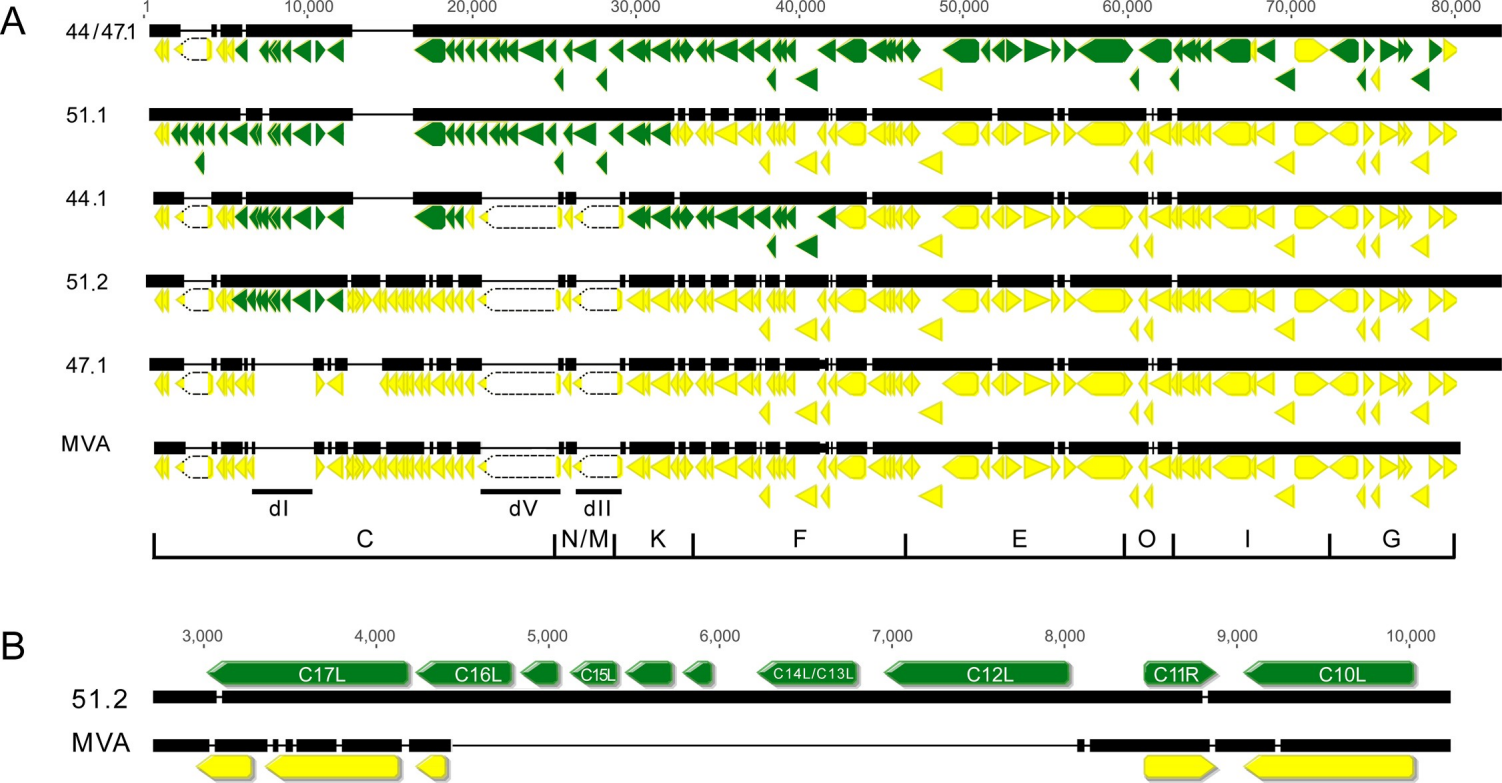
Multiple alignment of genome sequences of the left ends of MVA and HRE MVAs. **(A)** Alignment of left 80,00 bp. Arrows indicate the lengths and directions of ORFs. Solid yellow arrows, ORFs common to MVA; Green arrows, ORFs that differ from MVA by at least one nucleotide indicating they were derived by recombination from the cosmids; white arrows with dotted borders represent very small anomalous ORFs that are not to scale and connect the boundaries of deletions; dI, dV and dII indicate the sites of three of the large deletions in MVA. The corresponding *Hind*III fragments are shown below the alignments. **(B)** Alignment of the left 10,000 bp of MVA and v51.2. Names are provided for those ORFs annotated in the genome of the Copenhagen strain of VACV. C14L and C13L are separate ORFs in Copenhagen but a single ORF in v51.2.

The v51.2, v51.1, v44.1 and v44/47.1 HRE MVAs each replicated more than 3 logs higher than the parental MVA in MRC-5 cells (Fig 4A). However, even after deletion of C12L they still replicated at least one log higher than MVA suggesting the presence of one or more additional host range genes. The entire genomes of the recombinant HRE MVAs were sequenced in order to identify additional genes that might contribute to the alleviation of the host-range defect and were deposited in GenBank. In the multiple alignments depicted in Fig 6A, the ORFs derived from the partially overlapping cosmids used for marker rescue and have at least one nucleotide polymorphism are colored green and the ORFs identical to those in MVA are colored yellow. Inserted DNA was detected near the left ends of v51.1, v51.2, v44.1 and v44/47.1, consistent with the cosmids used for their generation. Although no DNA insertions were found anywhere in the v47.1 genome, there were some sequence differences presumably arising spontaneously that enabled replication in monkey but not human cells. The left end deletions I, V and II in MVA are annotated. Repair of deletion I, which included insertion of C12L, occurred in each of the recombinant viruses able to replicate in human cells, whereas repair of deletions II and V only occurred in v51.1 and v44/47.1 and therefore were not essential for replication although the F5L gene affects plaque morphology [26]. Furthermore, the genetic changes in v51.2, which were concentrated around deletion I, were present in each of the HRE MVAs capable of replicating in human cells (Fig 6A and 6B), suggesting that they included the minimal set of potential host range genes. Our strategy was to delete these genes from v51.2ΔSPI-1 to see if that further reduced virus spread in MRC-5 and A549 cells but not CEF.

**Fig 7:**
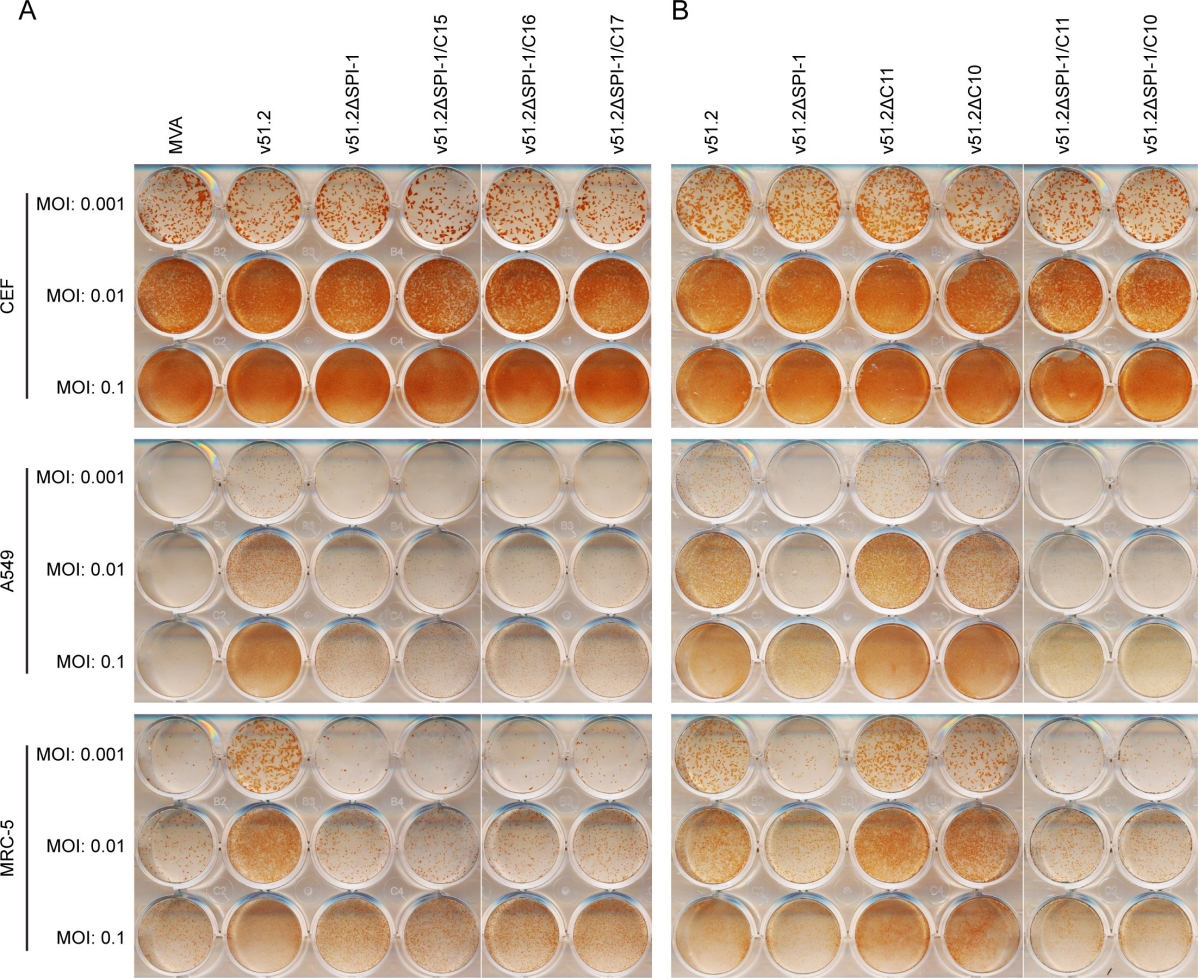
Effects of deletions of additional ORFs in v51.2 on virus spread. **(A, B)** CEF, A549 and MRC-5 cells in 12-well plates were infected with the indicated viruses at MOI of 0.001, 0.01 and 0.1 and overlaid with methylcellulose. After 48 h, the overlay was removed and cells were stained with antibody to VACV.

We focused on the C15L, C16L and C17L ORFs because they are absent in MVA but present in the HRE viruses. Therefore, these ORFs in the HRE viruses were individually deleted by replacement with mCherry regulated by the p11 promoter. MVA, v51.2, v51.2ΔSPI-1, v51.2ΔSPI-1ΔC15, v51.2ΔSPI-1ΔC16 and v51.2ΔSPI-1ΔC17 viruses replicated equally well in permissive CEF. In A549 and MRC-5 cells, the replication of v51.2 was diminished to the same extent by deletion of SPI-1 alone and deletion of both SPI-1 and either C15L, C16L or C17L (Fig 7A), suggesting that the latter genes are not involved in host range. Although C10L and C11R are present in MVA, there are sequence differences in the homologs of the HRE MVAs that potentially could affect host range. However, deletion of C10L or C11R from v51.2 or v51.2ΔSPI had no effect on virus spread in CEF or MRC-5 cells (Fig 7B), even though C11R is a growth factor [37, 38] and has been shown to enhance VACV spread under some conditions [39–41]. Thus, we did not identify an additional gene in v51.2 that significantly impaired replication in MRC-5 cells.

## Discussion

A previous study [24] showed that the host-range defect of MVA could be rescued by insertion of large DNA segments from the left end of the genome of a replication-competent virus. This result contrasted with the failure of the opposite approach: an attempt to generate the host-range defect in the CVA strain of VACV, which is the parent of MVA, by deleting DNA corresponding to the six major MVA deletions [22]. The failure of the latter strategy is somewhat perplexing as we showed here by genome sequencing that independently isolated HRE MVAs all had repaired deletion I and that one of these (v51.2) acquired no additional DNA. Moreover, deletion of SPI-1 located within the repaired deletion I segment of v51.2 restricted the host range. Therefore, if repair of deletion I can extend the host range of MVA and deletion of SPI-1 reduced the replication of HRE MVAs, why didn’t the introduction of deletion I into CVA restrict host-range? Synthetic lethality, a recognized genetic event in which deficiencies in the expression of two or more genes results in cell or organismal death whereas a deficiency in any one gene does not [42], could explain this discrepancy. Although this phenomenon is not usually considered for viruses because of their relatively small genomes, poxviruses have genomes encoding ~200 genes and evidence for synthetic lethality has been obtained in other contexts for VACV [43, 44]. Thus, a second gene that allows VACV replication in human cells could be present outside of the 6 deletions in CVA but not in MVA. The importance of the virus background received support from the fact that deletion of SPI-1 from RPXV is more debilitating than the same deletion from VACV WR. Also, the introduction of deletion I into the Lister strain of VACV reduced replication by 5-fold in HeLa cells (the reduction inMRC-5 cells was not presented) [23]. Therefore, deletion of SPI-1 causes no, moderate or severe host range defects depending on the orthopoxvirus strain.

Just as differences in the genetic background of VACV strains impact the effect of SPI-1 deletion, so do differences in human cell lines. Introduction of the SPI-1 ORF into MVA increased replication more in MRC-5 cells than HeLa and A549 cells. Nevertheless, deletion of the SPI-1 gene from an HRE MVA had a strong negative effect in A549 as well as in HeLa and MRC-5 cells. The cell-dependent differences in addition and deletion of SPI-1 suggests that there are two distinct mechanisms of host range restriction and that both are highly active in A549 cells but only one in MRC-5 cells. Thus, expression of SPI-1 is necessary for enhanced replication of MVA in MRC-5 and A549 cells but is sufficient only in the former.

The host-range function of SPI-1 was first revealed by the failure of spontaneous RPXV mutants to replicate in pig kidney and human A549 cells [29]. The major defect appeared as a block in virus particle maturation accompanied by some features of apoptosis [33]. We showed here that the aberrant particles that accumulate in cells infected with MVA and a VACV with a specific SPI-1 deletion look remarkably similar by electron microscopy. However, the host-range function of RPXV SPI-1 in A549 cells is abolished by mutation of the tryptophan to alanine in the predicted reactive loop [36], whereas the mutated SPI-1 retains host-range function for MVA in MRC-5 cells. This difference could be due to the presence of additional host factors that inhibit MVA or to differences in the mechanism of inhibition of RPXV and MVA SPI-1 deletion mutants. Taken together, these data also suggest that SPI-1 has more than one host range function depending on the virus and host cell backgrounds.

The present results are an important step towards the goal of understanding the basis for the human host range restriction and attenuation of a vector that forms the basis of numerous vaccines in clinical trials. In addition, the information could help to design new and improved vectors. Furthermore, the demonstration that human cells expressing SPI-1 support MVA replication may lead to the development of non-avian cell lines for propagation of candidate MVA vaccines. Finally, our finding that SPI-1 is host range factor for MVA can simplify use of high throughput RNAi or CRISPR/Cas single gene methods to identify additional viral and human restriction elements.

## Materials and methods

### Cells

A549 cells (ATCC CCL-185) were grown in Dulbecco’s modified Eagle’s medium/F-12 (Life Technologies) supplemented with 10% fetal bovine serum (FBS, Sigma-Aldrich), 2 mM L-glutamine, 100 units of penicillin, and 100 μg of streptomycin per ml (Quality Biologicals, Inc.). Primary CEF prepared from 10-day old fertile eggs (Charles River) and BS-C-1 (ATCC CCL-26) and MRC-5 (ATCC CCL-171) cells were grown in minimum essential medium with Earle’s balanced salts (EMEM) supplemented with 10% FBS, 2 mM L-glutamine, 100 units of penicillin, and 100 μg of streptomycin per ml (Quality Bologicals). HeLa cells (ATCC CCL-2) were grown in Dulbecco’s modified eagle’s medium (DMEM) supplemented with 10% FBS, 2 mM L-glutamine, 100 units of penicillin, and 100 μg of streptomycin per ml (Quality Biologicals).

### Antibodies

Rabbit antibody to VACV strain WR was described previously [45]; c-Myc antibody (9E10) conjugated to horse radish peroxidase (HRP) (catalog number sc-40 HRP) was from Santa Cruz Biotechnology; and rabbit anti-actin (catalog number A2066) was from Sigma-Aldrich.

### Viruses

WRΔSPI-1 was derived from the Western Reserve (WR) strain of VACV (ATCC VR-1354) and was described previously [31]. A panel of human replication-competent recombinant MVAs (v51.1, v51.2, v44.1, and v44/47.1) with segments of added VACV DNA of various lengths was described [24]. RPXV and VACV WR SPI-1 deletion mutants were described previously [31].

Modified viruses were constructed by homologous recombination using fluorescent reporter genes for selection. To generate MVA-SPI-1, a C12L DNA segment was introduced into the genome of MVA at the deletion III site by inserting the DNA fragment downstream of the mH5 promoter in pLW44-derived vector which also contains the P11 VACV promoter driven GFP [35]. The MVA-SPI-1 F322A and MVA-SPI-1 T309R were constructed by mutating the Phe322 into Ala and Thr309 into Arg using Q5 Site-Directed Mutagenesis Kit (New England Biolabs, Inc.).

C12L genes from v51.1, v51.2, v44.1, and v44/47.1 were deleted by homologous recombination with a PCR product containing the P11 VACV promoter-driven GFP gene flanked by sequences on either side of C12L. Fluorescent plaques were identified and cloned by repeated plaque isolation. Similarly, C10L and C11R were deleted by replacing the corresponding gene with P11 promoter-driven mCherry. Red plaques were picked and purified by repeated isolation. To generate vΔC12/C11 and vΔC12/C10, fluorescent foci that expressed both GFP and mCherry were picked and plaque purified. A similar strategy was adopted to delete the C15L, C16L, and C17L from v51.2ΔC12. The recombinant viruses were PCR amplified and sequenced to confirm the identities.

Homologous recombination was carried out by infecting CEF with 1 PFU/cell of virus, followed by transfection with assembled PCR products using Lipofectamine 2000 (Thermo Fisher). After 24 h, cells were harvested and lysed by three freeze-thaw cycles. The lysates were diluted 10-fold and used to infect CEF monolayers. Fluorescent recombinant plaques were distinguished from the parental plaques and clonally purified five times. The purities of the recombinant viruses were confirmed by PCR amplification and sequencing of the modified region. MVA and recombinant viruses were propagated in CEF.

### Virus yield determination

CEF were grown in 12-well plates and infected with 0.001 or 0.01 PFU/cell of virus in MEM supplemented with 2.5% FBS for 2 h. The cells were washed extensively with the same medium, incubated at 37°C, and harvested at 48 h after infection. Harvested cells were lysed by 3 freeze-thaw cycles, and virus titers were determined by plaque assay on CEF monolayers.

### Plaque assay and immunostaining

Virus samples were dispersed in a chilled water bath sonicator with two 30-s periods of vibration, followed by 10-fold serial dilutions in EMEM supplemented with 2.5% FBS. Diluted viruses were distributed onto CEF monolayers. After adsorption for 2 h, the medium was aspirated and replaced with medium containing 2.5% FBS and 0.5% methylcellulose. After 48 or 72 h, infected cells were fixed with methanol-acetone (1:1), washed with water, and incubated with rabbit anti-VACV antibody (1:2,000 dilution) for 1 h. The cells were washed again with water and incubated with a 1:3,000 dilution of protein A conjugated with peroxidase (Thermo Scientific) for 1 h. The cells were washed and incubated with the substrate dianisidine saturated in ethanol for 5 min. After color formation, the dianisidine solution was removed, and the cells were washed in water.

### Construction of the 2xMyc-SPI-1 cell lines

A549 and MRC-5 cells expressing the 2xMyc tagged SPI-1 protein were created using retroviral transduction. A eukaryotic codon-optimized SPI-1 ORF with an N-terminal 2xMyc tag (2xMyc-SPI-1) was inserted into pQCXIP (Clontech) to generate pQCXIP-2xMyc-SPI-1. Retrovirus particles were produced by co-transfecting pQCXIP or pQCXIP-2xMyc-SPI-1 (transfer plasmid), pMLV-Gag-Pol (packaging plasmid), and pVSV-G (VSV-G envelope plasmid) into 293T cells using Lipofectamine 2000. A549 and MRC-5 cells were infected with the retroviruses in the presence of 5 μg/ml polybrene (Sigma-Aldrich). The cells were subcultured and passaged several times in selection medium containing 1 μg/ml of puromycin (Sigma-Aldrich). The expression of SPI-1 protein was determined by Western blotting using HRP-conjugated anti-Myc antibody (9E10).

### Western blotting

Cells were harvested, washed, and lysed in Lysis buffer (20 mM Tris (pH 7.4), 150 mM NaCl, 2 mM EDTA, 1% Triton X-100 and protease inhibitor) on wet ice for 30 min with frequent agitation. Cell lysates were cleared by centrifugation at 13,000 xg for 10 min at 4°C; the proteins were resolved on 4 to 12% NuPAGE Bis-Tris gels (Thermo Fisher) and transferred to a nitrocellulose membrane with an iBlot2 system (Thermo Fisher). The membrane was blocked with 5% nonfat milk in Tris-buffered saline (TBS) for 1 h, washed with TBS with 0.1% Tween 20 (TBST), and then incubated with the primary antibody in 5% nonfat milk in TBST overnight at 4°C. The membrane was washed with TBST and incubated with the secondary antibody conjugated with horseradish peroxidase (Jackson ImmunoResearch) in TBST with 5% nonfat milk for 1 h. After the membrane was washed, the bound proteins were detected with SuperSignal West Dura substrates (Thermo Scientific).

### Transmission electron microscopy

The cells were fixed, dehydrated and embedded in Embed 812 resin (Electron Microscopy Sciences, Hatfield, PA) as described previously [46]. Specimens were viewed with a FEI Tecnai Spirit transmission electron microscope (FEI, Hillsboro, OR).

### Genome sequencing

Libraries for 454 pyrosequencing were made using Rapid Library Preparation Method Manual (October 2009) GS FLX Titanium Series (Roche, Branford, CT) and Paired End Library Preparation Method Manual, 3kb Span (October 2009) GS FLX Titanium Series. Each library was processed using emPCR Method, Manual Lib-L MV (October 2009) in separate emulsion reactions. The paired-end sample was loaded on a single lane and the fragment sample was loaded in two lanes of an 8-region 454 GS FLX Titanium sequencing run. Genome assembly and gap closure was performed as previously described [44]. Sanger sequencing was used to correct errors due to runs of identical nucleotides.

### Accession numbers

Genome sequences of MVA 44.1, 44/47.1, 47.1 and 51.2 were deposited as GenBank Accession Numbers MK314710, MK314711, MK314712 and MK314713, respectively. The GenBank Accession Number MG663594 for MVA 51.1 was previously published [44].
